# Comprehensive Analysis of a Novel Lipid Metabolism-Related Gene Signature for Predicting the Prognosis and Immune Landscape in Uterine Corpus Endometrial Carcinoma

**DOI:** 10.1155/2022/8028825

**Published:** 2022-02-12

**Authors:** Xiaofang Tan, Shuang Liu, Liangyu Yao, Guoliang Cui, Jinhui Liu, Jiayi Ding

**Affiliations:** ^1^Affiliated Maternity and Child Health Care Hospital of Nantong University, Nantong 226006, China; ^2^Department of Pathology, Sir Run Run Hospital of Nanjing Medical University, Nanjing, China; ^3^Department of Urology, First Affiliated Hospital of Nanjing Medical University, Nanjing 210029, Jiangsu, China; ^4^Department of Gastroenterology, Second Affiliated Hospital of Nanjing University of Chinese Medicine, Nanjing 210017, Jiangsu, China; ^5^Department of Gynecology, First Affiliated Hospital of Nanjing Medical University, Nanjing 210029, Jiangsu, China

## Abstract

Lipid metabolism is important in various cancers. However, the association between lipid metabolism and uterine corpus endometrial carcinoma (UCEC) is still unclear. In this study, we collected clinicopathologic parameters and the expression of lipid metabolism-related genes (LMRGs) from the Cancer Genome Atlas (TCGA). A lipid metabolism-related risk model was built and verified. The risk score was developed based on 11 selected LMRGs. The expression of 11 LMRGs was confirmed by qRT-PCR in clinical samples. We found that the model was an independent prediction factor of UCEC in terms of multivariate analysis. The overall survival (OS) of low-risk group was higher than that in the high-risk group. GSEA revealed that MAPK signaling pathway, ERBB signaling pathway, ECM receptor interaction, WNT pathway, and TGF-*β* signaling pathway were enriched in the high-risk group. Low-risk group was characterized by high tumor mutation burden (TMB) and showed sensitive response to immunotherapy and chemotherapy. In brief, we built a lipid metabolism gene expression-based risk signature which can reflect the prognosis of UCEC patients and their response to chemotherapeutics and immune therapy.

## 1. Introduction

As the second common gynecologic malignancy in women, UCEC has 65,620 new cases and 12,590 deaths in USA [1]. Additionally, in China, the five-year OS rate of UCEC is just over a half [2]. Comparing with the developed countries, China has a gap in the field of the UCEC treatment and survival rate [3]. For most common cancers, cancer survival has improved in the past 50 years except UCEC. Furthermore, death rates of UCEC increased over the past decade [1, 4]. Hence, it is crucial to explore the possible mechanisms of the development of UCEC.

As a new hallmark of malignancy, reprogramming of lipid metabolism has been demonstrated by accumulating evidence at present [5, 6]. The membrane biogenesis is upregulated in cancers; hence, lipogenesis is also strongly activated [7, 8]. Lipid uptake, storage, and lipogenesis are increased in various cancers [9–11]. Nath et al. found that enhanced free fatty acid uptake can promote the progression of hepatocellular carcinoma [12]. Moreover, blocking adipogenesis inhibits the growth of glioblastoma [13]. In 1996, Bershtein et al. reported that the levels of malonic dialdehyde, a by-product of lipids peroxidation, were slightly lower under basal conditions in tumor tissue [14].

Profiles of 519 UCEC patients were obtained from TCGA database. The LMRGs in UCEC and their correlation with the clinical information of UCEC were analyzed. Subsequently, we filtered 11 LMRGs and built a signature and then identified the prognosis capacity of the signature in training group, and the predictive accuracy of the signature was validated in testing group.

## 2. Methods

### 2.1. Dataset

We obtained RNA-seq data and normalized clinical profiles from TCGA (http://cancergenome.nih.gov/) database. The information of 552 UCEC samples and 35 normal samples was selected. In total, 548 samples with complete clinical data were extracted from 552 cases.

### 2.2. Selection of LMRGs

The LMRGs were enrolled from the Molecular Signature Database v5.1 (MSigDB) [15]. 614 LMRGs were obtained for further explore. Then, the different expressed LMRGs (DELMGs) between UCEC and normal tissues were screened with cut-off threshold (the adjusted FDR <0.05 and absolute |log2FC|> 1) via “Limma” package.

### 2.3. Consensus Clustering Analysis

The UCEC samples were assigned into two groups in terms of the expression of DELMGs with “Consensus Cluster Plus” in R. Then, we applied Kaplan–Meier and log-rank test to obtain the overall survival (OS) data. Chi-square test was carried out to calculate the age, histologic type, tumor status, stage, and grade of two clusters.

### 2.4. Generation and Prediction of Prognostic Signatures

We separated the entire patients into two groups randomly. 260 patients were screened as training group and 259 were chosen as testing group. Univariate analysis was utilized to screen potential prediction LMRGs in training group. For the purpose of avoiding overfitting effect, we excluded some highly associated genes through last absolute shrinkage and selection operator (LASSO) regression analysis. 13 LMRGs were filtered for multivariate regression analysis. In total, we identified 11 genes (LHB, FAAH, PLA2G4F, HPGDS, LRP2, PLA2G2A, CEL, CYP7B1, CCDC58, ACACB, and CH25H) to establish the signature. Risk score was evaluated according to the following formula:

(1) $$\begin{array}{c} \text{risk score}=\sum_{i=1}^{n}\text{coef}\left(i\right)\times x\left(i\right), \end{array}$$

where *n*, coef, and *x* mean the number of genes, the coefficient, and expression value. People were assigned into two groups in terms of median risk score of all samples. The correlation between OS and risk score varied by Kaplan-Meier and log-rank test. Receiver operating characteristic (ROC) curve and the area under the curve (AUC) were applied to evaluate the prognostic capacity of risk score through the “survivalROC” *R* package.

### 2.5. Verification of the Prognostic Signature

259 patients were screened as testing group. The patients in testing group were separated into two groups in the light of the same cut-off risk score. To assess the relationship between PFS and risk score, we used Kaplan–Meier curve and log-rank test. To estimate the prognosis ability of risk score, we completed ROC and AUC through “survivalROC” *R* package.

Then, the accuracy of the signature was verified in entire group. Kaplan–Meier analysis and ROC were employed. The Chi-square test was used to assess the relationship between the risk score and clinical factors in entire group. Univariate and multivariate regression analyses were applied to identify whether the signature was an independent prognosis element. We further verified the prediction value of the risk score using clinicopathological factors.

### 2.6. Establishment and Validation of a Nomogram

Nomogram containing all prognostic clinical factors was built. We then utilized calibration plots to analyze the reliability of the nomogram. The “rms,” “foreign,” and “survival” *R* package was applied to construct and validate the nomogram.

### 2.7. Total RNA Extraction and Quantitative Real-Time PCR (qRT-PCR)

RNA isolation of tissues was conducted by TRIZOL reagent (Thermo Fisher Scientific, USA) and cDNA was reverse-transcribed by Revert Aid First Strand cDNA Synthesis kit (Thermo Fisher Scientific, USA). Quantitative polymerase chain reactions for LHB, FAAH, PLA2G4F, HPGDS, LRP2, PLA2G2A, CEL, CYP7B1, CCDC58, ACACB, CH25H, and GAPDH were conducted in a volume of 20 *μ*l by SYBR-Green PCR kit (Takara, Tokyo, Japan). The expression of the genes was estimated through 2^−ΔΔCT^ method. The primer sequences are listed in Supplementary Table 1.

### 2.8. Gene Set Enrichment Analysis (GSEA)

GSEA can clarify whether the hallmark gene sets were differently enriched between the groups [15]. We conducted GSEA to compare survival differences between different risk groups in entire group. *p* < 0.05 and false discovery rate (FDR) < 0.25 were be supposed to be significantly different.

### 2.9. Tumor Mutational Burden (TMB) Analysis

The mutation data was extracted from TCGA and was analyzed using maftools. The TMB score was evaluated through the following formula:

(2) $$\begin{array}{c} \text{TMB }=\left(\frac{\text{total mutant bases}}{\text{total covered bases}}\right)\times 10^{6}. \end{array}$$

### 2.10. Evaluating Tumor-Infiltrating Immune Cells (TIICs)

CIBERSORT was utilized to assess the fraction of 22 immunocytes in the light of TCGA RNA-sequencing data. The immunoscore was evaluated by ESTIMATE algorithm through *R* “estimate” package. The threshold was *p* < 0.05.

### 2.11. Immune Prognostic Signature (IPS) Analysis

IPS can be obtained through machine learning method in the terms of four gene categories (PD1, PD-L1, PD-L2, and CTLA4) closely related to immune cells. IPS was assessed by z-scores of genes related to immunity which was extracted from the Cancer Immunome Atlas (https://tcia.at/home).

### 2.12. Immunotherapy Response Prediction

Immune Cell Abundance Identifier (ImmuCellAI) was employed to predict the response to immunotherapy [16], which can evaluate the abundance of 24 immunocytes and predict the effect of immunosuppressant.

### 2.13. The Response to Chemotherapy and Small Molecule Drugs

The curative effect of chemotherapy and small molecule drugs was assessed by a public database called Genomics of Drug Sensitivity in Cancer (GDSC, https://www.cancerrxgene.org). The half-maximal inhibitory concentration (IC50) was calculated which means the drug sensitivity.

### 2.14. Statistical Analysis

We analyzed statistical profiles by *R* 4.0.2. DELMGs between UCEC patients and controls were obtained by Wilcoxon's Test. We utilized Pearson's correlation coefficient to assess the relationships between LMRGs. Chi-square test was conducted to calculate the relationships between the risk score and clinicopathology factors. *p* < 0.05 was supposed to be different.

## 3. Results

### 3.1. DELMGs between UCEC Samples and Adjacent Normal Tissues

The brief workflow of this research was exhibited in the Supplementary Figure 1. We obtained 614 LMRGs for differentially expressed analysis. Then, the DELMGs between cancer and normal tissues were screened with our cut-off threshold (the adjusted FDR <0.05 and absolute |log2FC| > 1). Finally, 175 DELMGs were identified (Supplementary Table 2 and Supplementary Figure 2).

### 3.2. Consensus Clustering of LMRGs Distinguished Two Clusters of UCEC with Different Prognoses

**Figure 1:**
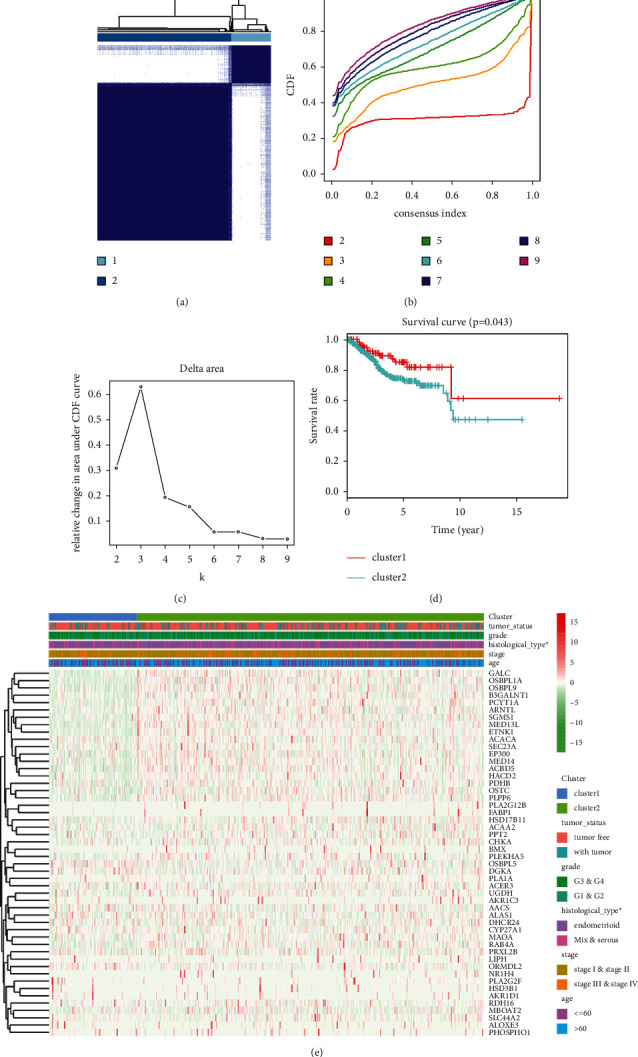
Differential clinicopathological features and survival of UCEC patients in two clusters in TCGA cohort. (a) TCGA UCEC group was assigned into two clusters when *k* = 2. (b) Consensus clustering cumulative distribution function (CDF) for *k* = 2 to 10. (c) Relative change in area under CDF curve for *k* = 2 to 10. (d) Kaplan-Meier OS curve for UCEC patients in the clusters. (e) The distribution of clinicopathological variables between different clusters.

**Figure 2:**
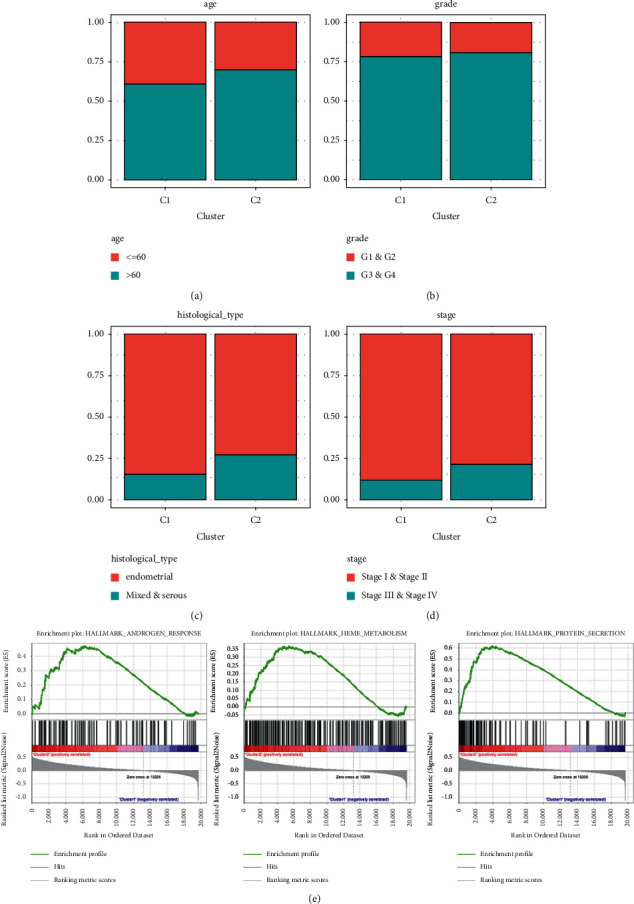
The distribution of clinicopathological variables between different clusters and GSEA analysis. (a) The distribution of ages between different clusters. (b) The distribution of grade between different clusters. (c) The distribution of histological type between different clusters. (d) The distribution of stage between different clusters. (e) GSEA was applied to analysis signaling pathways that were significantly enriched in patients in cluster 2.

*k* = 2 was identified to be an appropriate criterion to separate the UCEC patients into two subtypes, according to the expression similarity of DELMGs (Figures 1(a)–1(c)). A significant better OS was performed in cluster 1 (*p*=0.043) (Figure 1(d)). After that, we evaluated the relationship between the cluster and clinicopathological characteristics. We found that histologic subtype was not the same between clusters (Figure 1(e)). Patients in cluster 1 had younger age and lower grade and stage (Figures 2(a)–2(d)). Then, we conducted GSEA to explore the potential mechanisms (Figure 2(e)). Pathways like “androgen response,” “hememetabolism,” and “protein secretion” were enriched in cluster 2.

### 3.3. Consensus Clustering for LMRGs Associated with PD-L1, mRNAsi, and Immunocyte Infiltration

**Figure 3:**
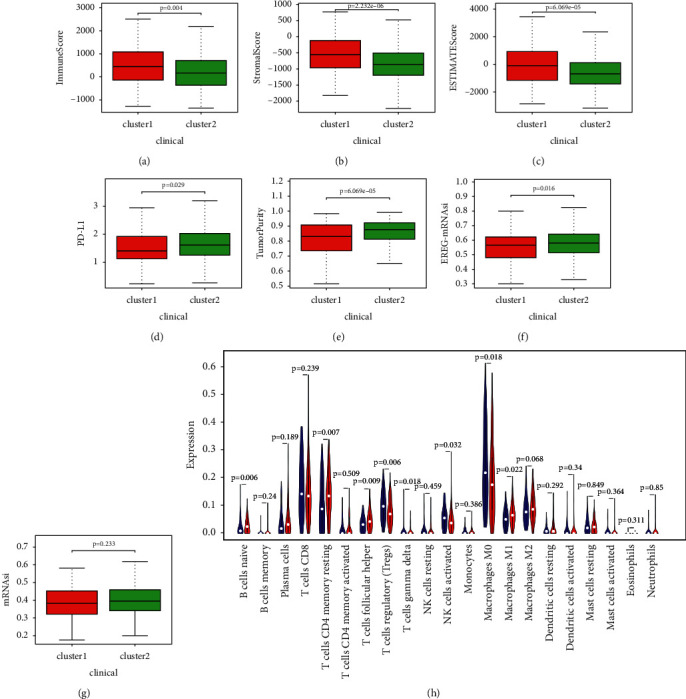
The relationship between cluster assignment and (a) immune score, (b) stromal score, (c) estimate score, (d) PD-L1, (e) tumor purity, (f) EREG-mRNAsi, and (g) mRNAsi. (h) The landscape of immune cell infiltration in UCEC.

In order to explore the relationship of estimated proportion of immune and stromal score with LMRGs, we calculated different scores of two subtypes. The immune, stromal, and estimate score were downregulated in cluster 2 (Figures 3(a)–3(c)). For investigating the involvement of PD-L1 and tumor purity with LMRGs, we identified differential expression status of PD- L1 and tumor purity in different clusters. The expression level of PD-L1 and tumor purity in cluster 1 were lower than those in cluster 2 (Figures 3(d)–3(e)). Additionally, by exploring the differences in mRNAsi and EREG-mRNAsi between two clusters, we found that patients in cluster 1 had lower mRNAsi and EREG-mRNAsi scores compared with patients in cluster 2 (Figures 3(f)–3(g)).

Subsequently, we assessed the immune infiltrate level between the clusters. The fraction of 21 immunocytes between different clusters was calculated. Cluster 2 had lower infiltration levels of regulatory T cells, gamma delta T cells, activated NK cells, and M0 macrophages, whereas cluster 2 had higher infiltration levels of some immune cells (Figure 3(h)).

### 3.4. Constructing the LMRGs-Related Risk Score in TCGA Training Group

After integrating mRNA expression profiles with OS data, we screened out 519 OS-related prognostic UCEC samples. The above EC samples were separated into training and testing group. In training group, we performed univariate regression analysis in terms of OS and then screened 24 genes which can predict the prognosis of UCEC, which met the standard that they were all closely related to OS (*p* < 0.05) (Supplementary Table 3). Then, LASSO regression analysis was applied to avoid overfitting situation (Supplementary Figures 3A–3B). Afterwards, we finally targeted 11 key genes (LHB, FAAH, PLA2G4F, HPGDS, LRP2, PLA2G2A, CEL, CYP7B1, CCDC58, ACACB, and CH25H) that met the modeling requirement (Supplementary Figure 3C) based on the multivariate Cox regression analysis. The risk scores of the training cohort were evaluated through the following formula:

(3) $$\begin{array}{c} \text{risk score }=\left(\text{LHB expression}\right)\times \left(-0.396\right)+\left(\text{FAAH expression}\right)\times \left(-0.0318\right)\\ +\left(\text{PLA}2\text{G}4\text{F expression}\right)\times \left(0.104\right)+\left(\text{HPGDS expression}\right)\times \left(-0.919\right)\\ +\left(\text{LRP}2\text{ expression}\right)\times \left(0.099\right)+\left(\text{PLA}2\text{G}2\text{A expression}\right)\times \left(0.166\right)+\left(\text{CEL expression}\right)\times \left(0.009\right)\;\\ +\left(\text{CYP}7\text{B}1\text{ expression}\right)\times \left(0.395\right)+\left(\text{CCDC}58\text{ expression}\right)\times \left(0.061\right)+\left(\text{ACACB expression}\right)\\ \times \left(0.516\right)+\left(\text{CH}25\text{H expression}\right)\times \left(0.046\right). \end{array}$$

**Figure 4:**
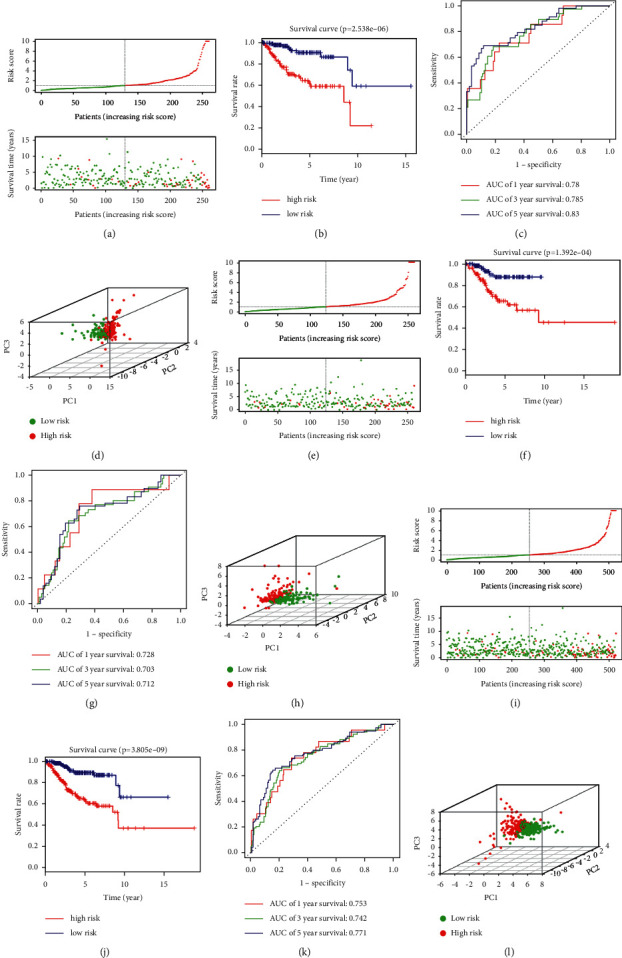
Survival analysis based on the risk model in training group. The distributions of risk scores and OS status in (a) training, (e) testing, and (i) entire group. The red and green dots represent the death and life. Kaplan–Meier plot shows that patients in high risk had significantly poorer OS than low-risk patients in (b) training, (f) testing, and (j) entire group. Time-dependent ROC curve analysis for survival prediction by the risk score in (c) training, (g) testing, and (k) entire group. Principal components analysis of whole gene expression data between two risk groups in (d) training, (h) testing, and (l) entire group.

In training group, samples were separated into two groups in terms of median risk score. The survival status of UCEC patients indicated that as the risk score increased, patients' mortality also increased, which meant worse prognosis (Figure 4(a)). Kaplan-Meier analysis performed that people with high-risk had worse prognosis than low-risk patients (Figure 4(b)). Time-dependent ROC analysis was utilized to assess the prediction capacity of the signature, in which the AUC was 0.78, 0.785, and 0.83 at 1,3, and 5 years separately (Figure 4(c)). All the work above showed that the signature was sensitive and accurate for prognosis prediction. PCA was applied to calculate the differential gene expression which means that patients in different groups tended to distribute differently (Figure 4(d)).

The univariate analysis revealed that the age (HR = 2.159, 95% CI = 1.121–4.160), stage (HR = 3.927, 95% CI = 2.156–7.153), histological type (HR = 2.412, 95% CI = 1.336–4.356), grade (HR = 2.662, 95% CI = 1.325–5.349), tumor status (HR = 13.037, 95% CI = 6.945–24.473), and risk score (HR = 1.067, 95% CI = 1.045–1.090) were related to OS (*p* < 0.05) (Supplementary Table 3). When using multivariate regression, the age (HR = 2.353, 95% CI = 1.136–4.875), tumor status (HR = 11.926, 95% CI = 5.851–24.307), and risk score (HR = 1.055, 95% CI = 1.031–1.081) were identified as the independent prediction parameters (*p* < 0.05) (Supplementary Table 3).

### 3.5. Verify the Prognostic Model in TCGA Testing Group

We validated the signature in testing group which included 259 patients and proved the same result as in training cohort (Figures 4(e)–4(h)). In testing group, survival status of UCEC patients showed that as the risk increased, patients' mortality increased, which meant worse prognosis (Figure 4(e)). High-risk patients were proved to have a poorer prognosis than low-risk people through Kaplan-Meier analysis (*p*=1.392*e* − 04) (Figure 4(f)). The time-dependent ROC curve was employed, and AUC was 0.728, 0.703, and 0.712 at 1, 3, and 5 years (Figure 4(g)). PCA was utilized to explore the different expression profiles between the risk groups in TCGA testing group which revealed that different groups seemed to distribute differently (Figure 4(h)).

The univariate analysis performed that the stage (HR = 4.775, 95% CI = 2.546–8.956), histological type (HR = 4.177, 95% CI = 2.235–7.805), grade (HR = 4.602, 95% CI = 1.933–10.957), tumor status (HR = 11.426, 95% CI = 5.707–22.875), and risk score (HR = 1.005, 95% CI = 1.001–1.009) (*p* < 0.05) were related to the OS (Supplementary Table 3). When these parameters were included into the multivariate regression, tumor status (HR = 6.773, 95% CI = 3.048–15.051) and risk score (HR = 1.005, 95% CI = 1.001–1.010) were considered as the independent prognostic elements (*p* < 0.05) (Supplementary Table 3).

### 3.6. Verify the Risk Score in TCGA Entire Group

We verified the prognostic signature in TCGA entire group and proved it in training group (Figures 4(i)–4(l)). The survival status of UCEC patients indicated that as the risk increased, the number of deaths increased, which meant bad outcome (Figure 4(i)). High-risk group was proved to have a poorer prognosis (*p*=3.805*e* − 09) (Figure 4(j)). The AUC was 0.753, 0.742, and 0.771 at 1, 3, and 5 years (Figure 4(k)). PCA was applied to evaluate the differential gene expression profiles between the groups in TCGA entire group which suggested that patients in the groups tended to distribute differently (Figure 4(l)). The different expression of 11 LMRGs between UCEC samples and normal tissues was shown in Supplementary Figure 4A. Furthermore, the correlation of 11 LMRGs was analyzed in Supplementary Figure 4B. Association between LMRGs expression and outcomes of UCEC patients was compared by analyzing the overall survival data of the TCGA UCEC cohort (Supplementary Figure 5).

**Figure 5:**
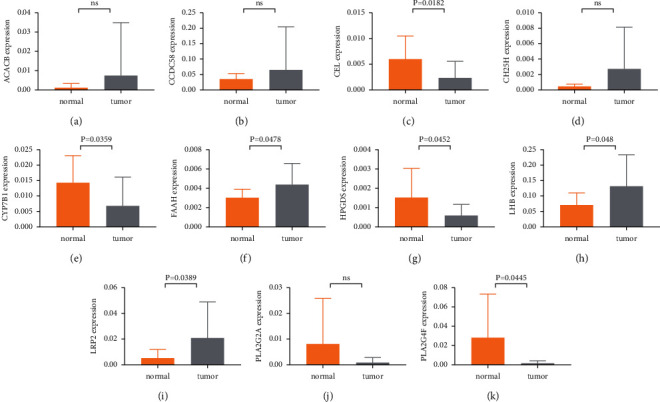
Expression level of (a) ACACB, (b) CCDC58, (c) CEL, (d) CH25H, (e) CYP7B1, (f) FAAH, (g) HPGDS, (h) LHB, (i) LRP2, (j) PLA2G2A, and (k) PLA2G4F in clinical samples.

In addition, the expression of 11 LMRGs was further validated by qRT-PCR in clinical samples (Figures 5(a)–5(k)). The results indicated that the mRNA expression of CEL, CYP7B1, FAAH, HPGDS, LHB, LRP2, and PLA2G4F was different between UCEC and normal samples. However, CEL and PLA2G4F expressions were contrary to the prediction from TCGA and there were no differences in ACACB, CCDC58, CH25H, and PLA2G2A expression between UCEC and normal tissues.

Univariate analysis performed that the age (HR = 1.788, 95% CI = 1.118–2.859), stage (HR = 4.070, 95% CI = 2.670–6.205), histological type (HR = 2.997, 95% CI = 1.972–5.835), grade (HR = 3.395, 95% CI = 1.975–10.957), tumor status (HR = 11.042, 95% CI = 7.048–17.300), and risk score (HR = 1.005, 95% CI = 1.002–1.008) were correlated to the OS (*p* < 0.05) (Supplementary Table 3). When these parameters were included into multivariate Cox regression, stage (HR = 1.719, 95% CI = 1.068–2.767), tumor status (HR = 7.887, 95% CI = 4.742–13.118), and risk score (HR = 1.004, 95% CI = 1.000–1.008) were considered as independent prognostic elements (*p* < 0.05) (Supplementary Table 3). The relationship between the risk score and clinical features was also calculated. Difference existed between the groups for the tumor status, grade, stage, and histologic subtype, while there was no significant difference for other parameters (*p* < 0.001) (Supplementary Figure 6). The differential expression of specific LMRGs between UCEC patients with different clinicopathological features was shown in Supplementary Figure 7.

**Figure 6:**
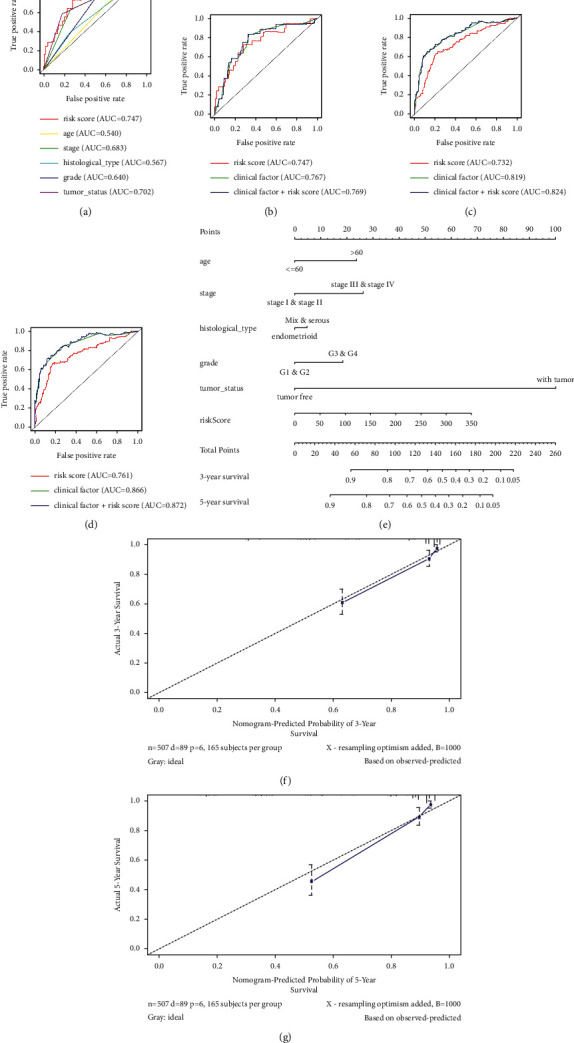
The time-dependent ROC curves for risk score and nomograms predicting survival probability of UCEC patients in TCGA. The time-dependent ROC curves for risk score and clinical factors combining with (a, b) 1-, (c) 3-, and (d) 5-year OS in TCGA UCEC cohort. (e) Nomogram to predict 3-year and 5-year OS. Calibration plots of (f) 3-year and (g) 5-year OS for nomograms.

ROC curve analysis of 1-year OS was utilized to demonstrate the prediction capacity of the risk score and clinicopathologic variables (Figure 6(a)). The 1-year AUC of the risk score (AUC = 0.747) was obviously higher than that of all clinicopathologic variables (Figure 6(a)). Additionally, ROC curve analysis of 1-, 3-, 5-year OS was utilized to demonstrate the prediction ability of the model, clinical factor, and clinical factor + risk score (Figures 6(b)–6(d)). The 1-, 3-, and 5-year AUC for risk score are obviously lower than those of clinical factor. However, when considering both clinical factors and risk score by Cox multivariate proportional hazards regression analysis, the 1-, 3-, 5-year AUC for clinical factor + risk score are much more favorable (Figures 6(b)–6(d)).

### 3.7. Establishment of UCEC OS Prediction Nomogram

Combining with clinicopathological characteristics related to prognosis, we built a nomogram for better predicting capacity of overall survival time (Figure 6(e)). We predict 3-year and 5-year survival rate of UCEC patients according to total points of all the factors. Calibration plots were used to perform the results consistently with previous results. Nomogram was proved to perform well for accurately predicting 3-year and 5-year survival rate of diagnosed patients (Figures 6(f)–6(g)).

### 3.8. Application of the Lipid Metabolism-Related Risk Score in Stratified Patients

To clarify the prognosis value of the model, patients were assigned into two groups according to clinical variables. As shown in Supplementary Figure 8, the OS rate was lower in high-risk people for the cases with age >60, age ≤60, grades 1-2, grades 3-4, patients with endometrioid carcinomas, those with mixed histological type, with stages I-II, or stages III-IV. All the *p*values were less than 0.05.

### 3.9. Functional Analysis of the Risk Score

**Figure 7:**
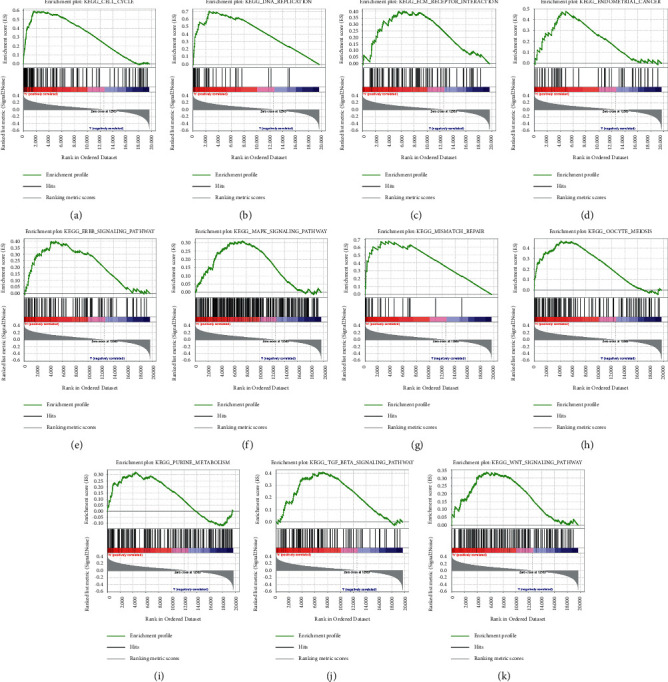
The enriched biological pathways: (a) cell cycle, (b) DNA replication, (c) ECM receptor interaction, (d) endometrial cancer, (e) ERBB signaling pathway, (f) MAPK signaling pathway, (g) mismatch repair, (h) oocyte meiosis, (i) purine metabolism, (j) TGF-beta signaling pathway, and (k) WNT signaling pathway from GSEA. ES, enrichment score; NES, normalized ES; NOM p-val, normalized *p*value.

GSEA was performed between tissues with different risk. We selected enriched biological pathways in terms of the normalized *p*value <0.05, FDR *q*-value <0.25, and normalized enrichment score. High risk was correlated to some pathways like cell cycle, DNA replication, ECM receptor interaction, endometrial cancer, ERBB signaling pathway, MAPK signaling pathway, mismatch repair, oocyte meiosis, purine metabolism, TGF-beta signaling pathway, and WNT signaling pathway (Figures 7(a)–7(k)).

### 3.10. Association of Tumor Mutation Burden (TMB) with Lipid Metabolism-Related Risk Score

**Figure 8:**
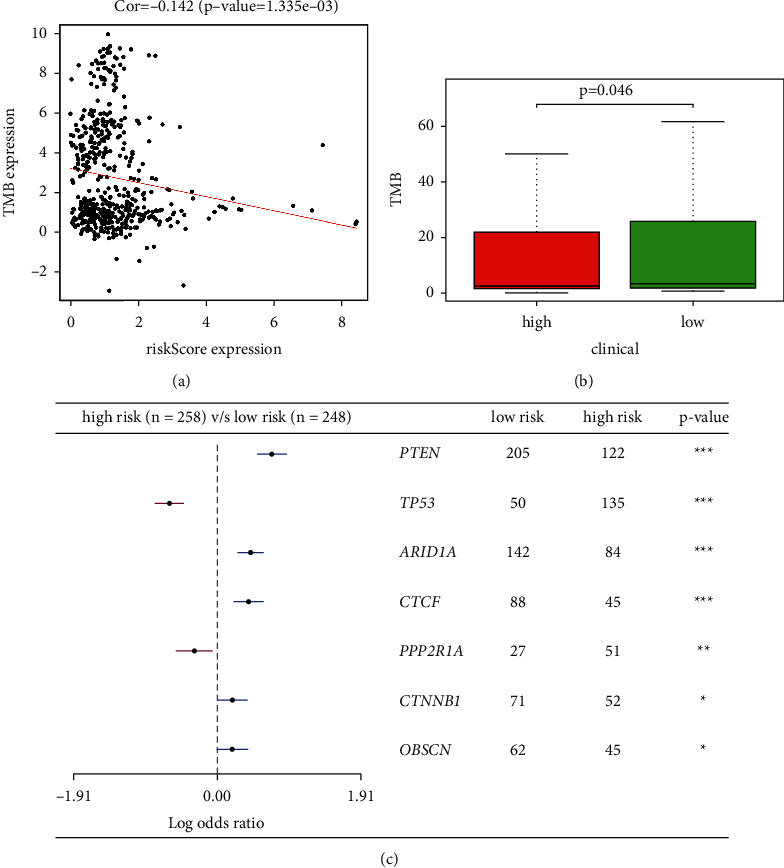
Association of TMB and specific mutated genes with risk score. (a) The correlation of TMB levels with risk score. (b) Lower TMB levels correlated with high-risk group (*p*=0.046). (c) The association of specific mutated genes with risk score.

Recently, several researches have showed that TMB was a predictive biomarker for immunotherapy in several cancers [17]. Patients with different TMB status showed distinct prognostic outcomes (Supplementary Figure 9A). Lipid metabolism-related risk score had negative correlation with TMB levels (*p*=1.335*e* − 03) (Figure 8(a)). Furthermore, TMB level was higher in low-risk people (*p*=0.046) (Figure 8(b)). Some specific mutated genes associated with the risk score were shown in Figure 8(c). Besides, combining the TMB status and risk score, patients could be separated into four groups and the prognostic outcome was different among these groups (Supplementary Figure 9B). Mutation information of genes was performed in waterfall chart, where, at the bottom, different colors represented different mutation types (Supplementary Figure 10). According to different classified categories, these mutations were further classified, among which missense mutation is occupying the most fraction (Supplementary Figure 10B). SNP occurred more frequently than the other two variant types (Supplementary Figure 10C), and *C* > *T* was the most common of SNV class in UCEC high-risk group (Supplementary Figure 10D). We calculated the number of altered bases; the result indicated the mutation type with different colors in box plot for the groups (Supplementary Figures 10E–F). Then, we exhibited the top 10 mutated genes in UCEC high-risk patients, which included TTN (36%), MUC16 (22%), CSMD3 (23%), PTEN (47%), PIK3CA (45%), KMT2D (27%), TP53 (52%), ARID1A (33%), TAF1 (22%), and PIK3R1 (28%) (Supplementary Figure 10G). However, the top 10 mutated genes in patients with high risk included TTN (42%), PTEN (83%), MUC16 (27%), ARID1A (57%), PIK3CA (52%), ZFHX3 (26%), PIK3R1 (34%), KMT2D (27%), CTCF (35%), and CTNNB1 (29%) (Supplementary Figure 11G). Mutation data of genes in low-risk group was performed in the waterfall chart (Supplementary Figure 11A). According to different classified categories, these mutations were further classified, in which missense mutation had the largest fraction (Supplementary Figure 11B). SNP occurred more frequently than other two variant types (Supplementary Figure 11C), and *C* > *T* was the most common of SNV class in UCEC low-risk group (Supplementary Figure 11D). We calculated the number of altered bases in each sample and performed the mutation type with different colors in box plot for UCEC low-risk group (Supplementary Figures 11E–F). Finally, we exhibited the top 10 mutated genes in UCEC high-risk group, which included TTN (42%), PTEN (83%), MUC16 (27%), ARID1A (57%), PIK3CA (52%), ZFHX3 (26%), PIK3R1 (34%), KMT2D (27%), CTCF (35%), and CTNNB1 (29%) (Supplementary Figure 11G).

### 3.11. Relation between m6A RNA Methylation and Lipid Metabolism-Related Risk Score

**Figure 9:**
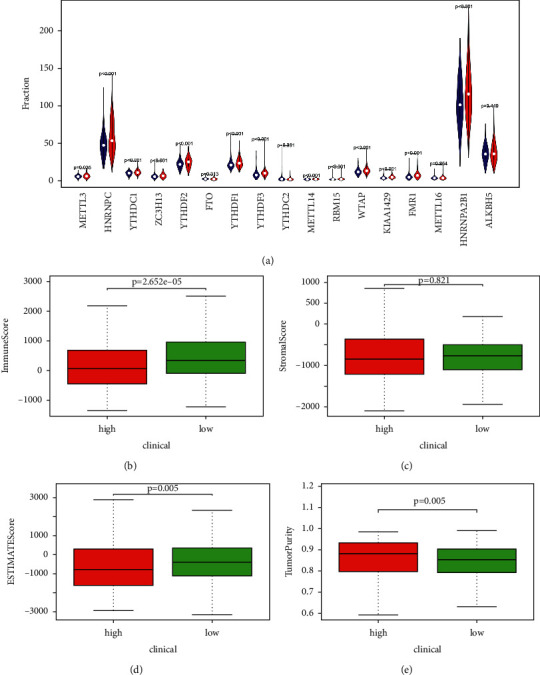
The landscape of m6A RNA methylation in UCEC and association of immune cell infiltration with lipid metabolism-related risk score. (a) The expression levels of 17 m6A RNA methylation regulators in two groups in the TCGA UCEC cohort. The red and blue indicated the high- and low-risk groups, respectively. (b) Immune score, (c) stromal score, (d) estimate score, and (e) tumor purity in the high- and low-risk groups.

Recently, some studies reported that m6A RNA methylation could regulate metabolic activity [5]. Hence, we explored the relation between m6A RNA methylation and lipid metabolism-related risk score in TCGA entire group. The expression levels of METTL3, METTL14, HNRNPC, HNRNPA2B1, YTHDC1, ZC3H13, YTHDF1, YTHDF2, YTHDF3, RBM15, WTAP, KIAA1429, and FMR1 were higher in high-risk patients (Figure 9(a)). The results performed the essential biological roles of m6A RNA methylation regulators possessed in lipid metabolism.

### 3.12. Connection between Distinct Immune Cell Infiltration and Lipid Metabolism-Related Risk Score

**Figure 10:**
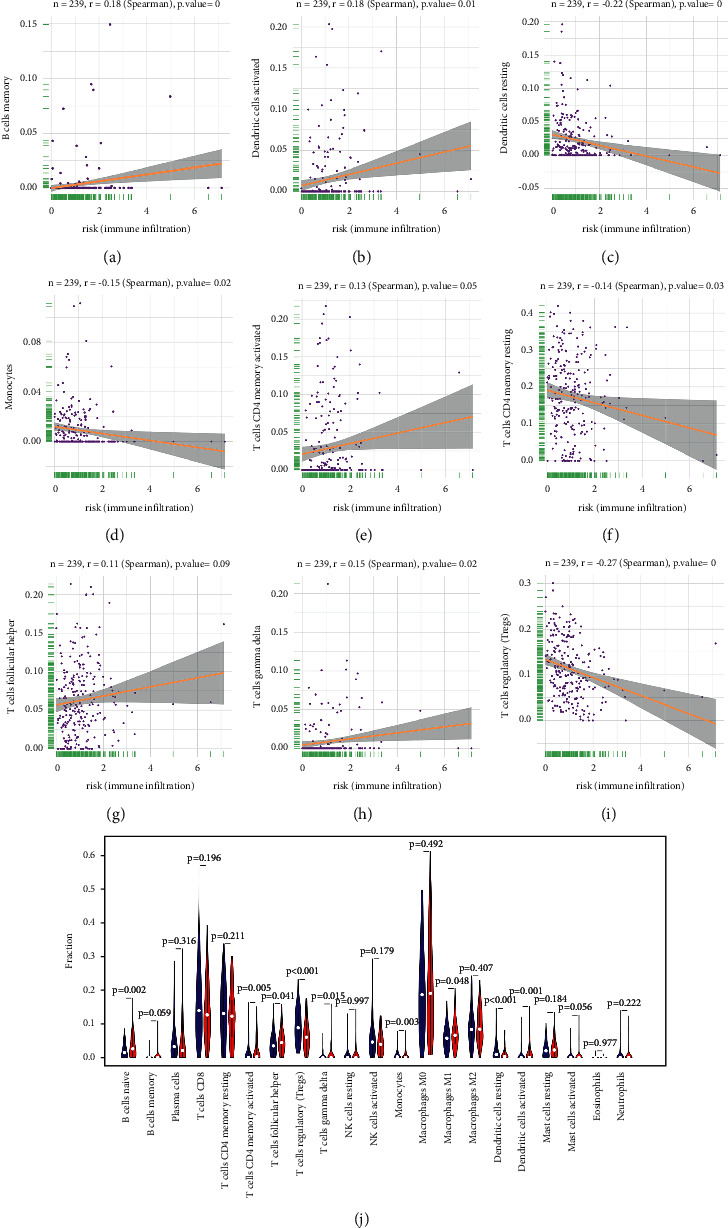
Correlation of TIICs proportion with risk score. Scattered plot showed the correlation of 9 kinds of tumor-infiltrating immune cells proportion: (a) B cells memory, (b) dendritic cells activated, (c) dendritic cells resting, (d) monocytes, (e) T cells CD4 memory activated, (f) T cells CD4 memory resting, (g) T cells follicular helper, (h) T cells gamma delta, and (i) T cells regulatory with the risk score (*p* < 0.05). Pearson's coefficient was applied to conduct correlation test. (j) Violin plot showed the ratio differentiation of 21 kinds of immunocytes between UCEC samples with risk score relatively to the median of risk score, and Wilcoxon rank sum was applied to significance test.

Recent studies revealed that metabolic pathway regulation is associated with immunology in cancers [18]. To clarify the effect of risk score in immune microenvironment of UCEC, we evaluated the immunoscore and immune infiltrate level. The immune, stromal, and estimate scores were downregulated in high-risk people (Figures 9(b)–9(e)). The fraction of 22 immune cell types between two subgroups was analyzed. In Figures 10(a)–10(i), risk score was positively associated with memory B cells, follicular helper T cells, activated Dendritic cells, gamma delta T cells, CD4 memory activated T cells, and monocytes, while it was negatively associated with regulatory T cells, CD4 memory resting T cells, resting Dendritic cells, and monocytes. These results revealed the connection between distinct immune cell infiltration and lipid metabolism-related risk score. Additionally, high-risk group showed higher infiltration levels of immune cells (Figure 10(j)).

### 3.13. Association of Immune Checkpoint Genes and mRNAsi with Lipid Metabolism-Related Risk Score

**Figure 11:**
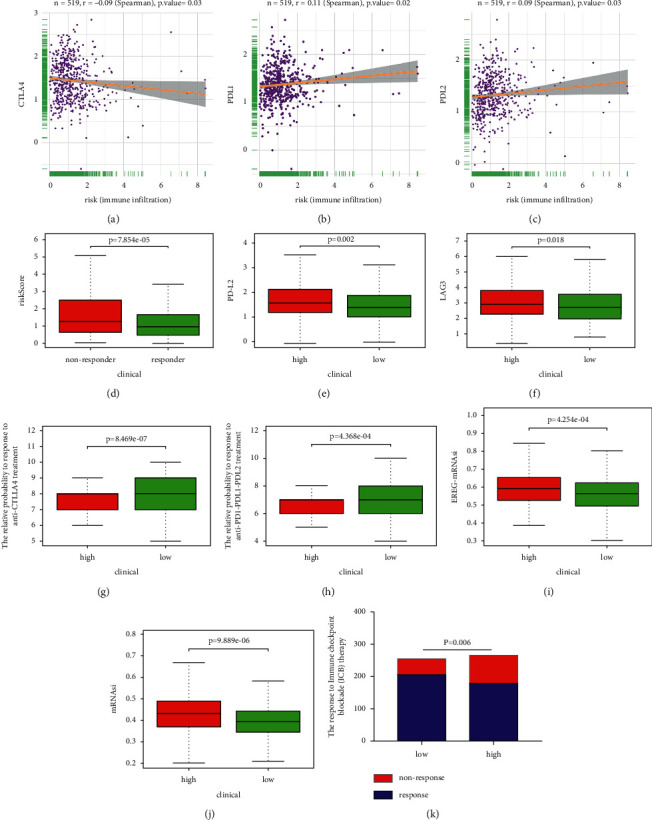
The relationship between risk score and (a–f) immune checkpoint molecules. (g, h) The relative probabilities to respond to immunotherapy. The association between risk score and (i) mRNAsi and (j) EREG-mRNAsi. (k) The prediction of immunotherapy response.

Immune checkpoint inhibitors have become a promising option in treating a variety of malignancies. In Figures 11(a)–11(d), risk score had negative relationship with the expression level of CTLA4 and was positively related to the expression level of PD-L1 and PD-L2. Besides, the expression levels of PD-L2 and LAG3 in high-risk group were high (Figures 11(e)–11(f)). According to the IPS analysis, the possibility of response to anti-PD-1/PD-L1/PD-L2 and anti-CTLA4 treatment were higher in low-risk patients (Figures 11(g) and 11(h)). MRNAsi (*p*=9.889*e* − 06) and EREG-mRNAsi (*p*=4.254*e* − 04) expression levels in patients with higher risk score were also higher (Figures 11(i)–11(j)). We further assessed the possibility of response to immunotherapy using the ImmuCellAI (Supplementary Table 4) and observed that patients in the low-risk group (80.71%, 205/254) were more likely to respond to immunotherapy (67.55%, 179/265; *p*=0.006; see Figure 11(k)).

### 3.14. Relationship of Chemotherapy and Small Molecule Drugs with Lipid Metabolism-Related Risk Score

**Figure 12:**
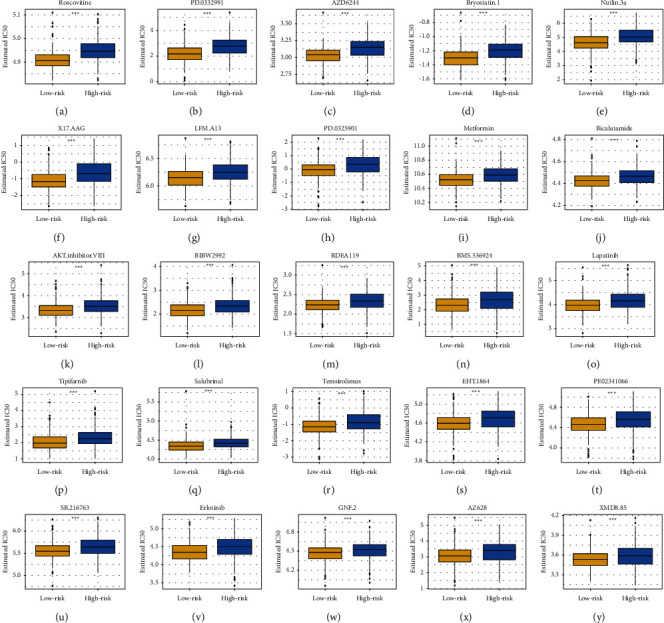
The estimated IC50 of immunotherapy drugs is higher in high-risk group. (a) Roscovitine, (b) PD.0332991, (c) AZD6244, (d) Bryostatin.1, (e) Nutlin.3a, (f) X17.AAG, (g) LFM.A13, (h) PD.0325901, (i) Metformin, (j) Bicalutamide, (k) AKT Inhibitor VIII, (l) BIBW2992, (m) RDEA119, (n) BMS.536924, (o) Lapatinib, (p) Tipifarnib, (q) Salubrinal, (r) Temsirolimus, (s) EHT.1864, (t) PF.02341066, (u) SB.216763, (v) Erlotinib, (w) GNF.2, (x) AZ628, and (y) XMD8.85. ^∗∗∗^*p* < 0.0001.

Given that chemotherapy and small molecule drugs are common method for treating UCEC cancer, GDSC database was used to evaluate the level of effect of commonly used chemotherapeutics and small molecule drugs. We assessed IC50 of each sample and there was a difference of IC50 between two risk groups among hundreds of drugs. Patients with low risk were more sensitive to these drugs (Roscovitine, PD.0332991, AZD6244, Bryostatin.1, Nutlin.3a, X17.AAG, LFM.A13, PD.0325901, Metformin, Bicalutamide, AKT Inhibitor VIII, BIBW2992, RDEA119, BMS.536924, Lapatinib, Tipifarnib, Salubrinal, Temsirolimus, EHT.1864, PF.02341066, SB.216763, Erlotinib, GNF.2, AZ628, and XMD8.85) (*p* < 0.0001, Figure 12).

## 4. Discussion

Lipids comprise a broad group of substances, which include fatty acids, sphingolipids, phospholipids, and triglycerides. As the second messengers, lipids can transmit signals within cells and provide energy when it is insufficient [7, 19]. Recent evidence revealed that the progression of various metabolic disease was correlated with dysregulation of lipid metabolism [20–22]. Extensive evidence suggested that reprogramming of lipid metabolism has a vital effect in cancer via energy production, signal transmission, and membrane synthesis [23, 24]. Besides, the effect of lipid synthesis inhibitors on anticancer was confirmed by preclinical studies and clinical trials [25, 26].

Compelling studies indicated that lipid metabolism contributed to various tumors, including breast cancer and glioblastoma [9, 27], whereas few studies reported the association between UCEC and lipid metabolism. In our research, the prognostic significance of LMRGs was explored in UCEC by evaluating the expression of DELMGs in UCEC. We selected 175 DELMGs among UCEC samples and adjacent normal tissues. From these 175 genes, we finally identified eleven (LHB, FAAH, PLA2G4F, HPGDS, LRP2, PLA2G2A, CEL, CYP7B1, CCDC58, ACACB, and CH25H) lipid metabolism gene-based risk signatures using LASSO and multivariate regression analysis. Studies reported that LHB is correlated with high-risk epithelial ovarian cancer and prostate cancer [28, 29]. It is also reported that FAAH expression level in endometrial cancer cell contributes to the regulation of plasma anandamide and N- palmitoylethanolamide concentrations in postmenopausal women suffering from endometrial cancer [30]. The relationship of HPGDS and colorectal cancer is also explored in recent study [31]. In gastric cancer, LRP2 was indicated to be a mutated gene by using next-generation sequencing technology [32]. PLA2G2A, a new beta-catenin/TCF target gene, can inhibit gastric cancer migration and invasion [33]. Moreover, high level of CEL was related to unfavorable outcome in breast cancer [34]. The hypermethylation of CYP7B1 is also identified to play vital roles in accumulation of 27-hydroxycholesterol in breast cancer [35]. 8 genes were identified which correlated with tumor progression in endometrial cancer using microarray gene expression analysis, which included CCDC58 [36]. Then, we analyzed the expression and prognosis relationship of the eleven genes in TCGA dataset.

ACACB, CH25H, CYP7B1, HPGDS, and PLA2G2A showed lower expression in UCEC samples compared with normal samples. On the contrary, the expression level of other six genes increased in UCEC, which indicated poor outcome. Furthermore, high expression of LHB, HPGDS, CEL, FAAH, and CH25H indicated unfavorable outcome, while high expression of PLA2G4F, LRP2, PLA2G2A, CYP7B1, CCDC58, and ACACB indicated better prognosis in UCEC.

To investigate the biological roles of these eleven genes in UCEC, GSEA was carried out; then we found that cell cycle, ECM receptor interaction, MAPK signaling pathway, ERBB signaling pathway, WNT signaling pathway, and TGF beta signaling pathway were involved in high-risk UCEC group. Increasing evidence showed that aberrant lipid metabolite can lead to the disorder of the immune system [37]. Some researchers have found complicated crosstalk between lipid metabolite and reprogramed immune cells like tumor-associated macrophages, T cells, and dendritic cells [38–41]. We also found a significant decrease in ESTIMATE indicating that the lipid metabolism is correlated with immunity status of UCEC.

We further analyzed if lipid metabolism gene-based risk signature could supply useful message about the reflection to immunotherapy and chemotherapy. The existing evidence has shown that some specific metabolic pathways are involved in immunotherapy response [42–44]. In this study, the results revealed that expression levels of PD-L1, PD-L2, and CTLA4 were associated with lipid metabolism gene-based risk signature. Besides, we observed that low-risk patients had sensitive response to immunotherapy than high-risk patients, indicating that high-risk patients might not benefit from immunotherapy.

Subsequently, by using GDSC dataset, patients with low-risk were more effective to chemotherapeutic or small molecule drugs such as Roscovitine, Bryostatin.1, Akt Inhibitor VIII, and Lapatinib. Interestingly, limited studies were reported to reveal the effects of these drugs on carcinoma. Roscovitine, a cyclin-dependent kinase (CDK) inhibitor, can inhibit cholangiocarcinoma cell in vivo [45]. Bryostatin.1, a macrolide lactone derived from marine organism *Bugula neritina*, is well known to suppress tumor in leukemia [46]. Besides, Bryostatin-1 may reduce HCC cells proliferation by promoting cyclinD1 proteolysis [47]. In addition, Akt Inhibitor VIII could benefit chemosensitization of cisplatin-resistant human oral squamous cell carcinoma by taking nimbolide synergistically. Akt-mediated regulation of the proapoptotic proteins Bax and caspase-3 has been proved in tumors and is related to drug resistance [48–50]. In fact, it has been demonstrated that alteration of protein kinase B (PKB/Akt) activity is crucial in human cancers [51]. Furthermore, Lapatinib may penetrate blood brain barrier to work on the brain. It was revealed to inhibit the progression of brain metastasis in breast cancer patients. These drugs with promising effect should be used in patients with low risk. However, high-risk patients may not benefit from these drugs.

However, there are still some deficiencies in our study. First, it only involves the LMRGs and did not involve other cancer related genes. After that, this study is based on bioinformatic analysis of online database; we need experimental research and large-sample clinical research to validate the predictive value of this signature.

## 5. Conclusions

Above all, based on eleven selected LMRGs, we developed a lipid metabolism-related gene expression-based risk score that can accurately predict the outcomes of UCEC patients. Low-risk people were related to favorable outcomes. Further research should be conducted to verify the risk signature.

## Data Availability

The data used to support the findings of this study are available upon request.

## Supplementary Materials

Supplementary Materials Supplementary Figure 1: the workflow of the research. Supplementary Figure 2: heatmap and volcano plots of UCEC patients from TCGA. Heatmap (A) and volcano (B) plots were generated with FDR < 0.05 and |log2FC| > 1, using the data of differentially expressed lipid metabolism-related genes in UCEC downloaded from TCGA. Supplementary Figure 3: prognostic value of lipid metabolism-related genes in TCGA UCEC training cohort. (A, B) Multivariate Cox regression via LASSO is presented, and ten candidate LMGs were selected in training cohort. (C) Forrest plot of the multivariate Cox regression analysis in UCEC. Supplementary Figure 4: the expression levels (A) and correlation (B) of 11 lipid metabolism‐related genes in UCEC tumor samples and adjacent normal pairs. ^*∗*^*p* < 0.05, ^*∗∗*^*p* < 0.01, and ^*∗∗∗*^*p* < 0.001. Supplementary Figure 5: prognostic evaluation of the 11 lipid metabolism-related genes in the entire TCGA UCEC cohort. Kaplan–Meier curves of OS in different expression levels of (A) LHB, (B) HPGDS, (C) CEL, (D) ACACB, (E) FAAH, (F) LRP2, (G) CYP7B1, (H) CH25H, (I) PLA2G4F, (J) PLA2G2A, and (K) CCDC58. Supplementary Figure 6: the heat map shows the expression of 11 lipid metabolism-related genes and the distribution of clinicopathological variables between the high- and low-risk groups. Supplementary Figure 7: the differential expression of specific lipid metabolism‐related genes and risk score between UCEC patients with different clinicopathological features. Supplementary Figure 8: prognostic value of the risk score in UCEC patients classified into specific cohorts. Kaplan-Meier survival curve of OS for patients with (A) age > 60, (B) age <= 60, (C) grades 1-2, (D) grades 3-4, (E) endometrioid, (F) mixed histological type, (G) stages I-II, and (H) stages III-IV. Supplementary Figure 9: the relationship with TMB, risk score, and patients' prognostic outcome. (A) TMB status and (B) combining TMB status and risk score. Supplementary Figure 10: landscape of mutation profiles and summary of the mutation information with statistical calculations in UCEC high-risk group. (A) Mutation information of each gene in each sample was shown in the waterfall plot, in which various colors with annotations at the bottom represented the different mutation types. The barplot above the legend exhibited the mutation burden. (A, B, and C) Classification of mutation types according to different categories is shown, in which missense mutation accounts for the most fraction, SNP showed more frequency than insertion or deletion, and C>T was the most common of SNV; (D, E) tumor mutation burden in specific samples is shown; (F) the top 10 mutated genes in UCEC high-risk group are revealed. Supplementary Figure 11: landscape of mutation profiles and summary of the mutation information with statistical calculations in UCEC low-risk group. (A) Mutation information of each gene in each sample was shown in the waterfall plot, in which various colors with annotations at the bottom represented the different mutation types. The barplot above the legend exhibited the mutation burden. (A, B, and C) Classification of mutation types according to different categories is shown, in which missense mutation accounts for the most fraction, SNP showed more frequency than insertion or deletion, and C > T was the most common of SNV; (D, E) tumor mutation burden in specific samples is shown; (F) the top 10 mutated genes in UCEC low-risk group are revealed. Supplementary Table 1: primer sequence of genes in qRT-PCR. Supplementary Table 2: the differentially expressed lipid metabolism‐related genes between UCEC tumor samples and adjacent normal pairs. Supplementary Table 3: univariate and multivariate Cox regression analyses of the prognosis-related factors. Supplementary Table 4: the response to immune checkpoint blockade therapy in 519 patients.
