# Supplementary material for: Comprehensive Analysis of a Novel Lipid Metabolism-Related Gene Signature for Predicting the Prognosis and Immune Landscape in Uterine Corpus Endometrial Carcinoma
Source: J Oncol. 2022 Feb 12;2022:8028825. doi: 10.1155/2022/8028825 (PMC8858058; doi:10.1155/2022/8028825)
Supplement: Supplementary Materials — Supplementary Figure 1: the workflow of the research. Supplementary Figure 2: heatmap and volcano plots of UCEC patients from TCGA. Heatmap (A) and volcano (B) plots were generated with FDR < 0.05 and |log2FC| > 1, using the data of differentially expressed lipid metabolism-related genes in UCEC downloaded from TCGA. Supplementary Figure 3: prognostic value of lipid metabolism-related genes in TCGA UCEC training cohort. (A, B) Multivariate Cox regression via LASSO is presented, and ten candidate LMGs were selected in training cohort. (C) Forrest plot of the multivariate Cox regression analysis in UCEC. Supplementary Figure 4: the expression levels (A) and correlation (B) of 11 lipid metabolism‐related genes in UCEC tumor samples and adjacent normal pairs. ∗p < 0.05, ∗∗p < 0.01, and ∗∗∗p < 0.001. Supplementary Figure 5: prognostic evaluation of the 11 lipid metabolism-related genes in the entire TCGA UCEC cohort. Kaplan–Meier curves of OS in different expression levels of (A) LHB, (B) HPGDS, (C) CEL, (D) ACACB, (E) FAAH, (F) LRP2, (G) CYP7B1, (H) CH25H, (I) PLA2G4F, (J) PLA2G2A, and (K) CCDC58. Supplementary Figure 6: the heat map shows the expression of 11 lipid metabolism-related genes and the distribution of clinicopathological variables between the high- and low-risk groups. Supplementary Figure 7: the differential expression of specific lipid metabolism‐related genes and risk score between UCEC patients with different clinicopathological features. Supplementary Figure 8: prognostic value of the risk score in UCEC patients classified into specific cohorts. Kaplan-Meier survival curve of OS for patients with (A) age > 60, (B) age <= 60, (C) grades 1-2, (D) grades 3-4, (E) endometrioid, (F) mixed histological type, (G) stages I-II, and (H) stages III-IV. Supplementary Figure 9: the relationship with TMB, risk score, and patients' prognostic outcome. (A) TMB status and (B) combining TMB status and risk score. Supplementary Figure 10: landscape of mutation profiles a [file 8028825.f1.zip › 8028825.f1/Supplement Table 1 (1).docx]

**Table 1 Primer sequence of genes in qRT-PCR**

|  | Forward sequence | Reverse sequence |
| --- | --- | --- |
| LHB | ATCTGTGCCGGCTACTGTCC | TCACGGTAGGTGCACACTGG |
| FAAH | GGGGACCTGGTCTCAATTCTG | CAATCACGGTTTTGCGGTACA |
| PLA2G4F | GGCGGGAAACCTACCCATAC | CCACAGTTGCACATAGCAGT |
| HPGDS | ACCAGAGCCTAGCAATAGCAA | AGAGTGTCCACAATAGCATCAAC |
| LRP2 | TATCCCTCGTGCTTATGTCTGT | TGAACTGGTAACCACCGCAG |
| PLA2G2A | ATGAAGACCCTCCTACTGTTGG | GCTTCCTTTCCTGTCGTCAACT |
| CEL | GTCACCTTCAACTACCGTGTC | GGCCGCGATATTCCTCTTCAC |
| CYP7B1 | ATGCAAGGATGGTCAGAAGTTTT | TGGGTGCCGCAGAAGATAATA |
| CCDC58 | AGTGGCGGTGTGAACTGTG | GCTGTTGGAACCGTAGTGTTTA |
| ACACB | GCCTCTGATAACTCAGGGGAG | CCAGTCCCGTTGGCTTGAA |
| CH25H | ATCACCACATACGTGGGCTTT | GTCAGGGTGGATCTTGTAGCG |
